# Supplementary material for: Nutrient Estimation from 24-Hour Food Recalls Using Machine Learning and Database Mapping: A Case Study with Lactose
Source: Nutrients. 2019 Dec 13;11(12):3045. doi: 10.3390/nu11123045 (PMC6950225; doi:10.3390/nu11123045)
Supplement: Supplementary file 1 [file nutrients-11-03045-s001.pdf]

Supplemental Material for

Nutrient estimation from 24-hour food recalls using machine learning and database mapping: a case study with lactose

Table S1. Examples of the ASA24 Food Name (FoodListTerm) and responses to ASA24 prompts, the corresponding ASA24 FoodCode and Food Description, and the output of the manual lookup into NDSR when searching the Food Name

Figure S1. Creation of NDSR User Recipes with embedded recipes

Figure S2. Creation of NDSR User Recipes for a "Not Futher Specified" (NFS) food

Table S2. Confidence ratings for the seven nutrients used to compare an ASA24 query to an NDSR match

Table S3. Examples of foods reported in ASA24 and the corresponding food from the manual lookup which are considered "high-confidence" and "low-confidence" matches

Figure S3. Principal Component Analysis (PCA) and t-Stochastic Neighbor Embedding (t-SNE) plots of the training foods

Table S4. The ASA24 FoodCode, Food Description, and ASA24 year for the five foods removed as outliers

Table S5. Performance metrics of the naïve baseline machine learning models

Table S6. Evaluation metrics for the machine learning models for the "high-confidence" test foods (n = 152)

Figure S4. Plots of the machine learning test results with and without "Salmon, raw"

Figure S5. Plots of the database matching training data results with and without outlier foods

Table S8. Evaluation parameters comparing the lactose from the manual lookup and the database-matching results for "high-confidence" foods

Table S10. The number of training and test foods correctly matched with the known FNDDS FoodLink for the database matching methods

Table S11. The number of lactose-free (0 g of lactose) ASA24-reported foods and the number of lactose-free Nutrient-Only and Nutrient + Text first matches

Tables S7 and S9 are supplied as separate files

Table S1. Examples of the ASA24 Food Name (FoodListTerm) and responses to ASA24 prompts, the corresponding ASA24 FoodCode and Food Description, and the output of the manual lookup into NDSR when searching the Food Name.

From *MS* or *Responses* files		From *INFMYPHEI* or *Items* files		From manual lookup
**Variable**	**Response**	**FoodCode**	**ASA24 Food Description**	**NDSR Description**
FoodListTerm	Yogurt (not frozen)	11411300	Yogurt, plain, nonfat milk	yogurt, plain, nonfat (<1% fat)
YogurtTypeMilk	Nonfat			
YogurtLoCalSwtnr	No			
YogurtFlav	Fruit variety (all flavors)			
YogurtUnitContainer	Cups			
YogurtPortionCup	1/2 cup			
AnythingAdded	No			
FoodListTerm	Sour cream	12310350	Sour cream, light	Sour cream, lowfat
SourCreamKind	Light			
SourCreamPortionSpoon	2 tablespoons			
FoodListTerm	Pizza	58106500	Pizza with meat, prepared from frozen, thin crust	pizza, frozen, with meat (e.g. sausage, pepperoni, or hamburger), thin crust, unknown if white or wheat crust
PizzaSource	Yes			
PizzaKind	Other- pepperoni cheese			
PizzaCrust	Thin			
PizzaSize	Large			
PizzaPortionPiece	More than 1 piece			
FoodListTerm	McDonald's Cheeseburger	27510310	Cheeseburger with tomato and/or catsup, on bun	McDonald's, lunch and dinner orders, cheeseburger
BurgerPortionSandwich	1 sandwich			

a

From *MS* or *Responses* files		From *INFMYPHEI* or *Items* files	
**Variable**	**Response**	**FoodCode**	**ASA24 Food Description**
FoodListTerm	Eggs	32130010	Egg omelet or scrambled egg, made with oil
EggPrep	Scrambled		
EggIngAdd	Yes		
EggOmeletCheese	No		
EggOmeletMeat	No		
EggOmeletVegetables	No vegetables		
EggFatPrep2	Other fat or oil		
EggUnit	Number of eggs		
EggSize	Large		
EggPortionNumber	More than 1 egg		

b

**FoodCode**	**SR code**	**SR description**	**Amount**	**Measure**	**Weight**
32130010	1123	Egg, whole, raw, fresh	2		100
32130010	11100000	Milk, NFS	2	TB	30.5
32130010	2047	Salt, table	0.125	TS	0.75
32130010	82101000	Vegetable oil, NFS	1.5	TS	6.813

c

1. Egg omlet or scrambled egg, made with oil ASA24 2016 - 32130010
   1 servings made (serving = 100.0 grams)
   Components/Ingredients:
   1.i1 eggs, whole, raw
   72.43070 G (1.45 large)
   1.i2 Milk NFS ASA24 2016 - 11100000 (Milk NFS2)
   0.2209136 servings eaten (serving = 100.0 grams)
   1.i3 salt, regular
   0.54323 G (0.09 TS)
   1.i4 Vegetable oil, NFS ASA24 2016 - 82101000 (Veg oil NFS2)
   0.0493470 servings eaten (serving = 100.0 grams)

Figure S1. Creation of NDSR User Recipes with embedded recipes. a) The ASA24 Food Name and Food Descriptions were retrieved from subject *MS* (ASA24-2014) or *Responses* (ASA24-2016) files. Note: Certain ASA24 Food Descriptions contain multiple foods. b) The corresponding FNDDS recipe for FoodCode 32130010 retrieved from the USDA Food Composition Standard Reference database (SR). Note: Each 8-digit SR Code in the recipe refers to additional recipes. c) NDSR User Recipes were created following the ingredient list, additional recipes, and amounts shown in b).

a)

From *MS* or *Response* files		From *INFMYPHEI* or *Items* files	
**Variable**	**Response**	**FoodCode**	**ASA24 Food Description**
FoodListTerm	Milk (unknown type)	11100000	Milk, NFS
MilkPortionAddition	1/4 cup		

b)

**Food code**	**SR code**	**SR description**	**Amount**	**Measure**
11100000	1077	Milk, whole, 3.25% milkfat, with added vitamin D	31	GM
11100000	1079	Milk, reduced fat, fluid, 2% milkfat, with added vitamin A and vitamin D	38	GM
11100000	1082	Milk, lowfat, fluid, 1% milkfat, with added vitamin A and vitamin D	14	GM
11100000	1085	Milk, nonfat, fluid, with added vitamin A and vitamin D (fat free or skim)	17	GM

c)

1. Milk NFS ASA24 2016-11100000
   1 servings made (serving = 100.0 grams)
Components/Ingredients:
   1.i1 milk, whole (3.5 - 4% fat)
      31 G (0.13 CP)
   1.i2 milk, 2% fat or reduced fat
      38 G (0.16 CP)
   1.i3 milk, 1% fat or lowfat
      14 G (0.06 CP)
   1.i4 milk, skim, nonfat or fat free
      17 G (0.07 CP)

Figure S2. Creation of NDSR User Recipes for an NFS food. a) The ASA24 Food Name and Food Descriptions. This Food Description stated, "Not Further Specified" (NFS) since the food was of "unknown type" b) The corresponding FNDDS recipe for FoodCode 11100000 was retrieved from the USDA Food Composition Standard Reference database (SR). Note: Food descriptions with "NFS" are often a mix various types of the same food in one recipe. c) The NDSR User Recipe output. The ingredient list and amount were guided by b).

Table S2. Confidence ratings for the seven nutrients used to compare an ASA24 query to an NDSR match

Nutrient	Low	Medium[1]	High
kcal	≥ 128	85 -128	≤ 85
Total Protein	≥ 10	5 - 10	≤ 5
Total Fat	≥ 3	2.5 - 3	≤ 2.5
Total Carbohydrate	≥ 20	10 - 20	≤ 10
Calcium	≥ 200	100 - 200	≤ 100
Phosphorous	≥ 200	100 - 200	≤ 100
Sodium	≥ 200	100 - 200	≤ 100

The values represent the absolute difference between the two foods. A "low" confidence meant large nutrient value difference above the cut off, "medium" confidence meant nutrient value difference fell between the given range, and a "high" confidence level meant a small nutrient value difference that was below the cut off.

[1]Ranges are non-inclusive

Table S3. Examples of foods reported in ASA24 and the corresponding food from the manual lookup that are considered "high confidence"[1] and "low confidence" matches.

ASA24 FoodCode	ASA24 Food Description	NDSR Food Description	Food Description Confidence Level	Nutrient Confidence Level
High-Confidence Examples				
**11112110**	Milk, cow's, fluid, 2% fat	milk, 2% fat or reduced fat	H	7
**14104100**	Cheese, Cheddar	Cheddar cheese, unknown type	H	7
**11111000**	Milk, cow's, fluid, whole	milk, whole (3.5 - 4% fat)	H	7
**11531000**	Eggnog, made with whole milk	eggnog, regular	H	5
**53347000**	Pie, pumpkin	pies, pumpkin	H	6
**58100800**	Enchilada, just cheese, meatless, no beans, red-chile or enchilada sauce	enchiladas, without beans, cheese (no meat)	H	6
**52202060**	Cornbread, made from home recipe	cornbread, prepared from recipe	H	7
**55101000**	Pancakes, plain	pancake, plain or buttermilk, unknown type	H	6
**92101920**	Blended coffee beverage, made with regular coffee, milk, and ice, sweetened	frappuccino, unknown type, regular	H	7
**58106725**	Pizza with meat and vegetables, regular crust	pizza, from homemade recipe or restaurant, with one meat topping (sausage, pepperoni or hamburger), with vegetables, without extra cheese, thick crust or deep dish	H	6
Low-Confidence Examples				
**81302070**	Pesto sauce	pesto sauce, unknown if commercial or homemade	H	4
**58100300**	Burrito with beans and rice, meatless	Taco Bell, lunch and dinner orders, burritos, black bean	L	6
**14502040**	Imitation cheese	American cheese, process	L	3
**58107100**	Pizza, no cheese, thick crust	pizza, crust, white, thick	M	2
**95120010**	Nutritional drink or meal replacement, high protein, ready-to-drink, NFS	special formulated products, drinks, meal replacement drink, unknown if regular or low calorie	M	6

[1]A match was considered "high confidence" if at least five of the individual nutrients used to evaluate the match were rated as "high", and if the food description confidence level was rated as "high". The nutrients and rating cut-offs are in Table S2. The food description confidence level was a based on the manual lookup team's assessment of how similar the prompts were between the ASA24 and NDSR systems and how similar the output food descriptions were.

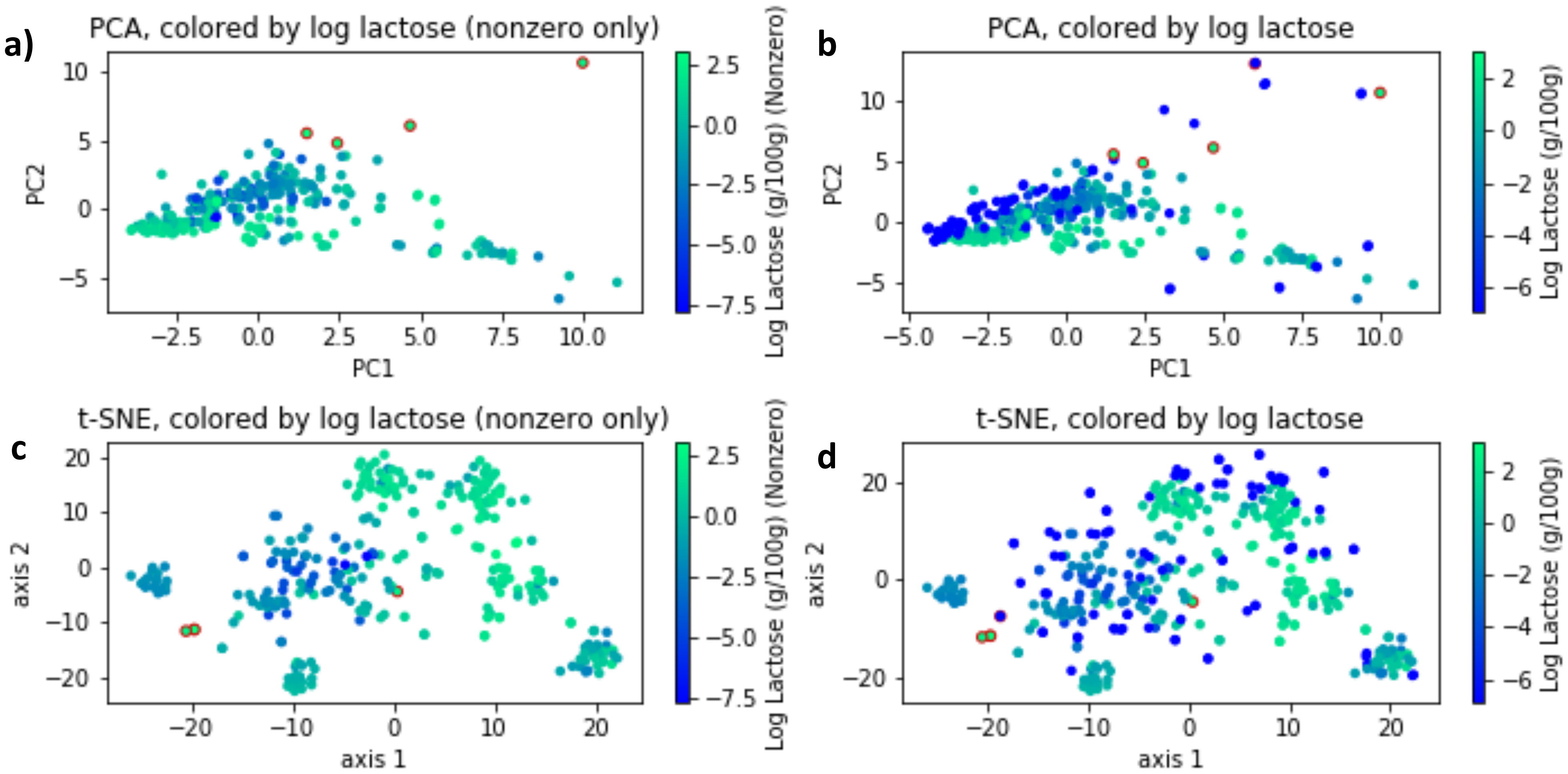

Figure S3. a) Principal Component Analysis (PCA) scores plot of the training foods with > 0 g of lactose, b) PCA scores plot of all training foods, c) t-distributed stochastic neighbor embedding (t-SNE) plots of the training foods with > 0 g of lactose, and d) t-SNE plots of all the training foods. Markers outlined in red indicate outliers as determined by *scikit-learn's IsolationForest*. The 62 nutrients and ASA24 year were used as input. A value of 0.001 was added to all lactose values to allow for log transformation.

Table S4. The ASA24 FoodCode, Food Description, and ASA24 year for the five foods removed as outliers[1]

FoodCode	Food_Description	ASA24 Year
11830800	Instant breakfast, powder, not reconstituted	2014
41430310	Protein diet powder with soy and casein	2014
43102110	Sunflower seeds, hulled, roasted, without salt	2014
95201000	Carnation Instant Breakfast, nutritional drink mix, regular, powder	2016
95220010	Nutritional drink mix or meal replacement, high protein, powder, NFS	2016

[1]outliers were determined by *scikit-learn's IsolationForest*.

Table S5. Performance metrics of the naïve baseline machine learning models

Performance Metric	Mean	Median	Median Non-zero	Perfect Classifier + Mean Regressor
**R^2**	-0.02	-0.32	-0.08	0.13
**SRC**	0	0	0	0.73
**PCC**	0	0	0	0.41
**MAE**	1.94	1.68	1.77	1.53

Spearman's rank correlation coefficient (SRC); Pearson's correlation coefficient (PCC); mean absolute error (MAE)

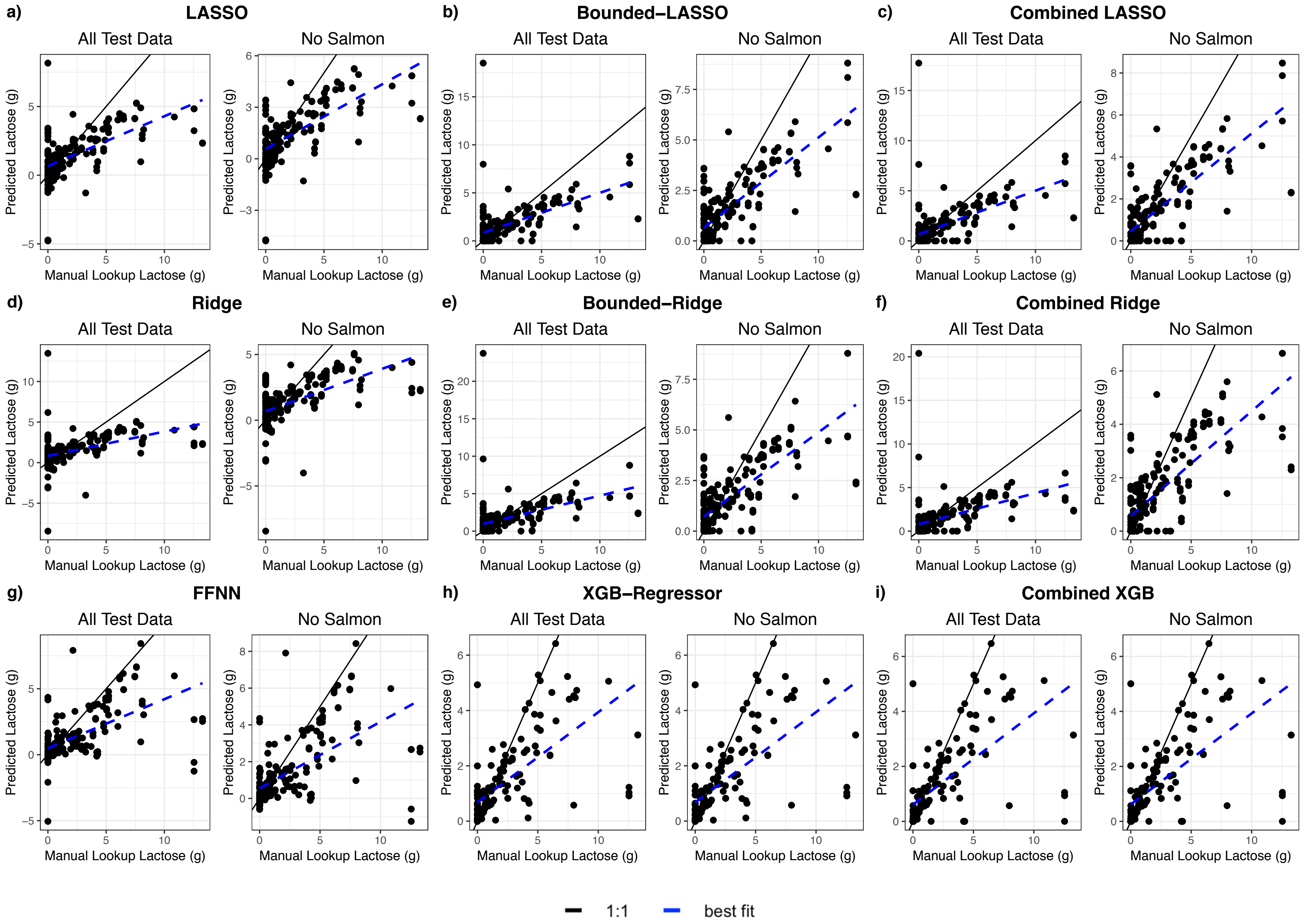

Figure S4. Plots of the machine learning test results comparing predicted lactose to the manual lookup lactose value with (left panels) and without (right panels) "Salmon, raw" (ASA24 FoodCode 26137100) for the a) LASSO, b) Bounded-LASSO, c) Combined LASSO, d) Ridge, e) Bounded-Ridge, f) Combined Ridge, g) Feed Forward Neural Network (FFNN), h) XGB-Regressor, and i) Combined XGB models.

Table S6. Evaluation metrics for the machine learning models for the "high confidence"[1] test foods (n = 152)

	**LASSO**	**Bounded-LASSO**	**Combined LASSO**	**Ridge**	**Bounded-Ridge**	**Combined Ridge**	**FFNN**	**XGB-Regressor**	**Combined XGB**
R^2	0.40	0.47	0.49	0.40	0.47	0.48	0.52	*0.53*	*0.53*
SRC	0.68	0.68	0.77	0.68	0.69	0.78	0.72	0.77	*0.78*
PCC	0.68	0.73	0.77	0.68	0.73	0.77	0.77	*0.79*	0.78
MAE	1.31	1.15	1.07	1.32	1.16	1.09	1.02	0.96	*0.92*
Classifier Accuracy	NA	NA	0.89	NA	NA	0.89	NA	NA	*0.92*

[1]A "high confidence" food has a "high" text matching rating and at least five of the seven individual nutrients (Total kcal, Total Protein, Total Carbohydrate, Calcium, Phosphorous, or Sodium) were in high confidence between the ASA24 query food and the NDSR match from the manual lookup process.

SRC: Spearman Rank Coefficient; PCC: Pearson's Correlation Coefficient; MAE: Mean Absolute Error

Italicized values indicate the highest R^2, SRC, PCC, and Classifier Accuracy, and the lowest MAE.

Table S7 is supplied as a separate file.

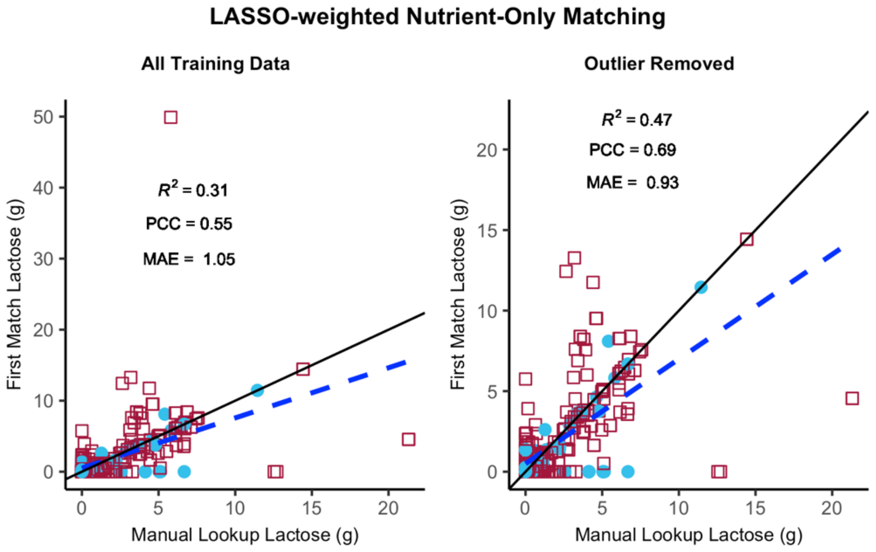

Fig S5. Plots of the database matching training data results with and without outlier foods for the a) LASSO-weighted Nutrient-Only matching, b) LASSO-weighted Nutrient + Text matching, and c) Ridge-weighted Nutrient + Text Matching. Pearson's correlation coefficient (PCC); Mean absolute error (MAE).

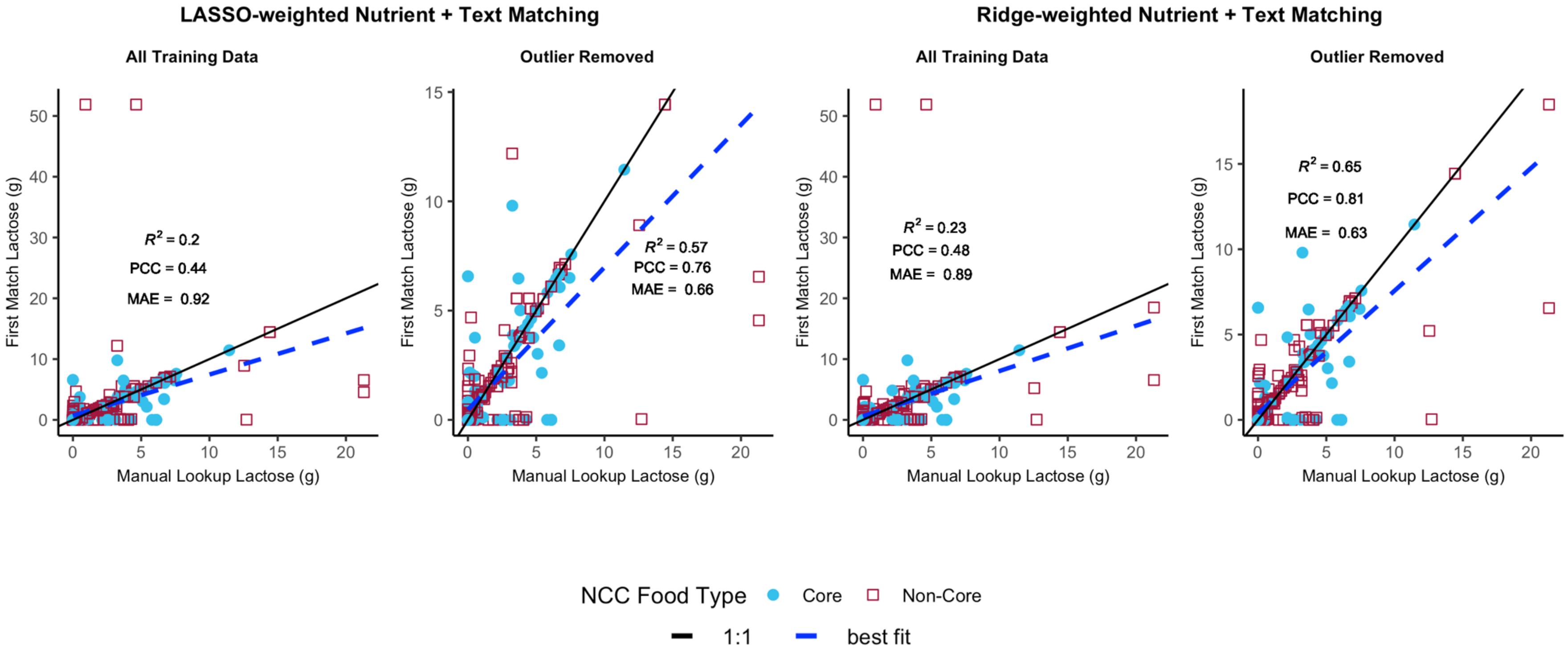

Table S8. Evaluation parameters comparing the lactose from the manual lookup and the database-matching results for "high confidence" foods

**Matching Algorithm**	**Weighting**	**Training (n = 339)**				**Test (n=152)**				**All Data (n = 491)**			
		**R^2**	**PCC**	**MAE**	**Variation**	**R^2**	**PCC**	**MAE**	**Variation**	**R^2**	**PCC**	**MAE**	**Variation**
**Nutrient-Only**	**Unweighted**	0.78	0.88	*0.53*	*0.35*	0.46	0.68	*0.97*	*0.50*	0.66	0.81	*0.67*	*0.38*
	**LASSO-weighted**	0.42 (0.67)	0.65 (0.82)	0.90 (0.77)	0.65 (0.65)	0.33	0.58	1.20	0.68	0.37 (0.54)	0.60 (0.74)	1.00 (0.91)	0.67 (0.67)
	**Ridge-weighted**	*0.80*	*0.89*	0.56	0.44	*0.51*	*0.71*	1.02	0.53	*0.69*	*0.83*	0.70	0.48
**Nutrient + Text**	**Unweighted**	*0.85*	*0.92*	*0.38*	*0.48*	0.64	0.80	0.68	0.53	*0.77*	*0.88*	*0.46*	0.51
	**LASSO-weighted**	0.24 (0.77)	0.49 (0.88)	0.77 (0.48)	0.52 (0.52)	0.63	0.80	0.69	0.56	0.27 (0.70)	0.52 (0.84)	0.76 (0.56)	0.54 (0.54)
	**Ridge-weighted**	0.24 (0.76)	0.492 (0.872)	0.77 (0.49)	0.50 (0.50)	*0.68*	*0.83*	*0.59*	*0.52*	0.28 (0.72)	0.53 (0.84)	0.73 (0.64)	*0.50 (0.50)*

A "high confidence" food has a "high" text matching rating and at least five of the seven individual nutrients (Total Kcal, Total Protein, Total Carbohydrate, Calcium, Phosphorous, or Sodium) were in high confidence between the ASA24 query food and the NDSR match from the manual lookup process. The R^2 and MAE are comparisons of the g of lactose between the manual lookup and the first match from the matching algorithm. The variation represents the median coefficient of variation in g of lactose among the top 5 matches returned by the matching algorithm. Italicized values indicate the highest R^2, lowest MAE, and lowest variation for the Nutrient-Only and Nutrient + Text algorithms for a given dataset.

Table S9 is supplied as a separate file

Table S10. The number of training and test foods correctly matched with the known FNDDS FoodLink the Nutrient-Only and Nutrient + Text database matching.

Dataset	Weighting		Nutrient Only		Nutrient + Text	
		Total n Foodlinks	n as 1st match	n in top 5 matches	n as 1st match	n in top 5 matches
Training	Unweighted	33	13 (39.4%)	23 (69.7%)	19 (57.6%)	30 (90.9%)
	LASSO		9 (27.3%)	16 (48.5%)	18 (54.5%)	28 (84.8%)
	Ridge		9 (27.3%)	19 (57.6%)	18 (54.5%)	28 (84.8%)
Test	Unweighted	9	3 (33.3%)	4 (44.4%)	5 (55.6%)	6 (66.7%)
	LASSO		1 (11.1%)	4 (44.4%)	5 (55.6%)	6 (66.7%)
	Ridge		2 (22.2%)	4 (44.4%)	5 (55.6%)	6 (66.7%)

Table S11. The number of lactose free (0 g of lactose) ASA24-reported foods and the number of lactose-free Nutrient-Only and Nutrient + Text first matches and the number where the first five matches were all lactose free.

**Dataset**	**Weighting**		**Nutrient Only**	**Nutrient + Text**
		**0-lactose manual matches**	**first match is 0-lactose**	**first match is 0-lactose**
Training	Unweighted	87	65 (74.7%)	70 (80.5%)
	LASSO		61 (70.1%)	68 (78.2%)
	Ridge		65 (74.7%)	69 (79.3%)
Test	Unweighted	44	37 (84.1%)	38 (86.4%)
	LASSO		30 (68.2%)	37 (84.1%)
	Ridge		38 (86.4%)	38 (86.4%)
